# Thyroid Transcription Factor 1 Reprograms Angiogenic Activities of Secretome

**DOI:** 10.1038/srep19857

**Published:** 2016-02-25

**Authors:** Lauren W. Wood, Nicole I. Cox, Cody A. Phelps, Shao-Chiang Lai, Arjun Poddar, Conover Talbot, David Mu

**Affiliations:** 1 Leroy T. Canoles Jr. Cancer Research Center, Eastern Virginia Medical School, Norfolk, VA 23501, USA; 2 Department of Microbiology and Molecular Cell Biology, Eastern Virginia Medical School, Norfolk, VA 23501, USA; 3 Institute for Basic Biomedical Sciences, The Johns Hopkins School of Medicine, Baltimore, MD 21205, USA.

## Abstract

Through both gain- and loss-of-TTF-1 expression strategies, we show that TTF-1 positively regulates vascular endothelial growth factor (VEGF) and that the *VEGF* promoter element contains multiple TTF-1-responsive sequences. The major signaling receptor for VEGF, i.e VEGFR2, also appears to be under a direct and positive regulation of TTF-1. The TTF-1-dependent upregulation of VEGF was moderately sensitive to rapamycin, implicating a partial involvement of mammalian target of rapamycin (mTOR). However, hypoxia did not further increase the secreted VEGF level of the TTF-1^+^ lung cancer cells. The TTF-1-induced VEGF upregulation occurs in both compartments (exosomes and exosome-depleted media (EDM)) of the conditioned media. Surprisingly, the EDM of TTF-1^+^ lung cancer cells (designated EDM-TTF-1^+^) displayed an anti-angiogenic activity in the endothelial cell tube formation assay. Mechanistic studies suggest that the increased granulocyte-macrophage colony-stimulating factor (GM-CSF) level in the EDM-TTF-1^+^ conferred the antiangiogenic activities. In human lung cancer, the expression of *TTF-1* and *GM-CSF* exhibits a statistically significant and positive correlation. In summary, this study provides evidence that TTF-1 may reprogram lung cancer secreted proteome into an antiangiogenic state, offering a novel basis to account for the long-standing observation of favorable prognosis associated with TTF-1^+^ lung adenocarcinomas.

Approximately 70% of lung adenocarcinomas (ADs) are positive for the expression of a lung development master regulator, thyroid transcription factor-1 (TTF-1 or known as NKX2-1)^1^. Thus, TTF-1 is routinely used by pathologists to differentiate lung ADs from the TTF-1^−^ squamous cell carcinomas of the lung and to identify lung ADs from nonpulmonary, nonthyroid tumors^2^. Because TTF-1 expression status is frequently analyzed in the clinics for human lung cancer, any new understanding of TTF-1 biology will likely inspire follow-up research to improve clinical practices. The notion of TTF-1 functionally contributing to lung tumorigenesis was founded on the discoveries by us^3^ and others^4^,^5^,^6^ that it is recurrently amplified in human lung cancer genomes. Although *TTF-1* gene amplification suggests a prooncogenic role^7^,^8^, later studies^9^,^10^,^11^,^12^,^13^ repeatedly detected antitumorigenic/antimetastatic activities of *Ttf-1* with the protumorigenic function of *Ttf-1* only manifested in specific genetic contexts^10^.

Our laboratory has been investigating the biology of *TTF-1* since our original discovery of its gene amplification in lung cancer^3^. We first explored the connection of *TTF-1* to microRNAs (miRNAs) and uncovered the miRNAs that regulate or are regulated by *TTF-1*^14^,^15^, placing *TTF-1* in a miRNA-based signaling network^7^. Next, we detected that the epithelial tight junction factors, *OCCLUDIN* and *CLAUDIN-1*, are direct transcriptional targets of TTF-1^11^. This observation, plus the reported finding that *E-CADHERIN* is also a transcriptional target of TTF-1^16^, warrants active research to tease out how various lung epithelial junctional structures are controlled by TTF-1 and the associated functional consequences in lung cancer and physiology. More recently, inspired by our interest to understand how TTF-1 would impact the secreted proteome (proteinaceous secretome), we conducted a focused screening for cytokine expression alterations in response to TTF-1 upregulation. VEGF stood out from this profiling exercise because in humans the lung exhibits the highest VEGF concentration which is 500 times higher than in plasma^17^. It has been proposed that the high levels of VEGF protein on the respiratory epithelial surface may function as a physiological reservoir^17^. Curiously, TTF-1^+^ alveolar type II (ATII) epithelial cells are generally considered the major source of VEGF in the lung^18^,^19^,^20^,^21^. However, a direct regulatory relationship between *TTF-1* and *VEGF* was never established, despite the fact that genetic perturbation of *Ttf-1* alters the expression of Vegf in animal systems^22^,^23^. By using both gain- and loss-of-TTF-1 expression strategies, we establish that *VEGF* is likely a direct target of TTF-1. Surprisingly, the conditioned media (CM) of TTF-1-overexpressing (and thus VEGF-enriched) lung cancer cells shows an inhibitory activity in the endothelial cell tube formation assay which scores angiogenicity. Further mechanistic characterizations reveal that a surge of GM-CSF in the CM of TTF-1^+^ lung cancer cells may be the culprit for the negative angiogenic phenotype of the CM of TTF-1^+^ lung cancer cells. Hence, our study establishes yet another venue to investigate the biology of the multi-faceted, lung development and cancer gene *TTF-1*, i.e. the secretome.

## Results

### TTF-1 Alters the Expression Profiles of Cytokine and Angiogenic Factors of Lung Cancer Cells

**Figure 1:**
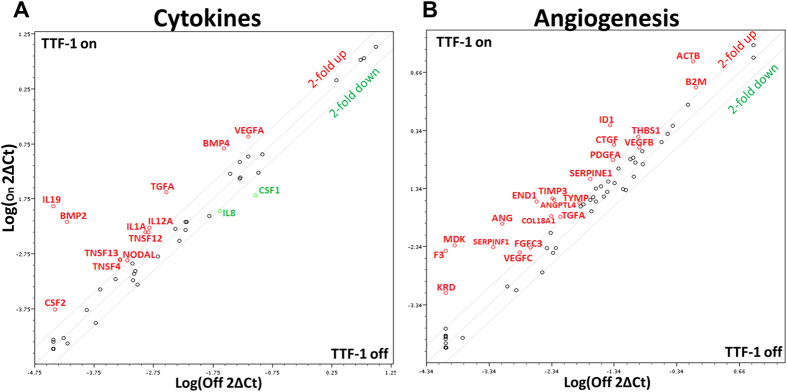
TTF-1 alters expression of cytokines and angiogenic factors. Focused RT-qPCR expression profiling of 84 cytokine (**A**) or angiogenic factors (**B**) was conducted using commercial qPCR arrays (Qiagen). RNAs representing TTF-1-on/off states were isolated from a TTF-1-inducible cell system based in the premalignant BEAS-2B cells in which a *TTF-1* transgene could be turned on by doxycycline (dox).

In our previous studies^11^,^15^, we created a doxycycline (dox)-inducible, wt-*TTF-1*-expressing cell system based in the non-malignant lung epithelial cell line, BEAS-2B. To explore how wt-*TTF-1* (referred to as *TTF-1* hereafter) modulates lung cancer secretome, we used a commercial qPCR array that targets 84 cytokines (Qiagen) to profile the RNA expression changes of the TTF-1 inducible system before and after turning on *TTF-1* expression. Notably, we detected an increase in the levels of *BMP4* (5.3X) and *VEGFA* (3.5X) (Fig. 1A). Since *BMP4* and *VEGFA* are functionally linked to angiogenesis, we surmised that *TTF-1* may regulate other angiogenic factors. To test our hypothesis, we conducted a second qPCR array profiling with the *TTF-1* inducible BEAS-2B cells using an angiogenesis-focused qPCR array targeting 84 angiogenic factors (Qiagen). The results were surprising in that most of the angiogenic factors that showed expression perturbation upon turning on *TTF-1* expression moved in the direction of upregulation (Signed Rank Test, *p* < 0.001, 21 upregulated >2-fold without any factor showing >2-fold downregulation, Fig. 1B).

### Detection of Increased VEGF Secretion in Additional Lung Cancer Cells

**Figure 2:**
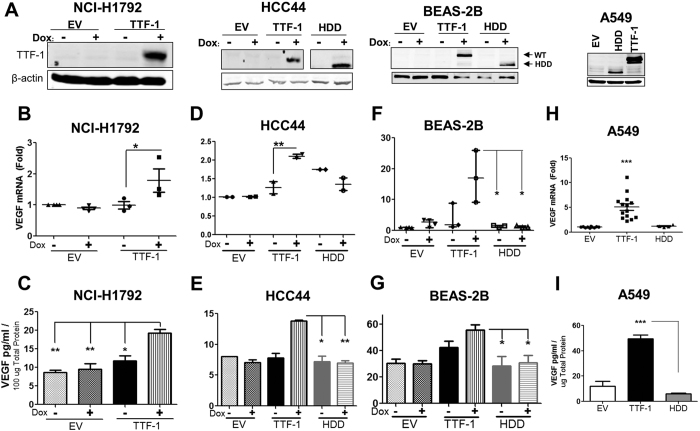
TTF-1 upregulates VEGF. Three dox-on TTF-1-inducible cells systems were hosted in two human lung AD cell lines (NCI-H1792 and HCC44) and a premalignant human lung epithelial cell line (BEAS-2B). A dox-on transgene of the HDD mutant of *TTF-1* was also created in HCC44 and BEAS-2B cells. (**A**) TTF-1^−^ human lung AD cell line (A549) was used to host stable expression of either wt-*TTF-1* or HDD mutant transgene. Protein expression of the dox-on *TTF-1* transgene is shown in *A*, whereas the *VEGF* RNA (**B**,**D**,**F**,**H**) of the transfectant cells and the secreted VEGF protein in the corresponding CM (**C**,**E**,**G**,**I**) demonstrate *VEGF* upregulation by TTF-1. EV, empty vector.

Motivated by the fact that *VEGFA* is a *bona fide* master regulator of angiogenesis^24^, we focused the subsequent studies on investigating the putative regulatory relationship between *TTF-1* and *VEGFA* (the two terms, VEGF and VEGFA, are referred to interchangeably in this study). We first used ELISA to quantify VEGF level in the CM of two additional TTF-1 inducible cell systems based in human lung AD cell lines (NCI-H1792 and HCC44) as well as the BEAS-2B-based system. The *TTF-1* transgene induction by dox was verified by immunoblotting (Fig. 2A). Two *VEGF* specific qPCR primer sets were used to demonstrate an increase in *VEGF* RNA in the cells treated with dox for 48 hr - NCI-H1792 (Fig. 2B), HCC44 (Fig. 2D), and BEAS-2B (Fig. 2F). Secreted VEGF within the concentrated CM was significantly elevated in all three *TTF-1* inducible cell lines (Fig. 2C,E, and G). As controls, two cell lines (BEAS-2B and HCC44) were also modified to express the homeodomain deleted (HDD) mutant^11^ of *TTF-1* upon dox induction. The levels of VEGF in HDD expressing cells did not increase concomitantly with the induction of the HDD mutant expression. Finally, we interrogated the cell lysates and CM of the A549 cell stably expressing TTF-1 or controls (HDD or EV) for *VEGF* RNA and protein level and detected a robust increase in both species in a TTF-1-dependnet manner (Fig. 2H,I). Moreover, we used a Luminex technology-based platform (Bio-Plex MAGPIX, Biorad) to quantify VEGF levels in the CMs of A549-based stable transfectant cells. The VEGF levels in the CM of A549-EV or A549-HDD cells were ~300 pg/mL, whereas the VEGF concentration in the CM of A549-TTF-1 cells was ~2000 pg/mL (data not shown). So far, the forward, gain-of-TTF-1 expression data are in line with the notion that TTF-1 upregulates *VEGF* transcription and secretion.

### Depletion of TTF-1 Lowers VEGF Secretion

**Figure 3:**
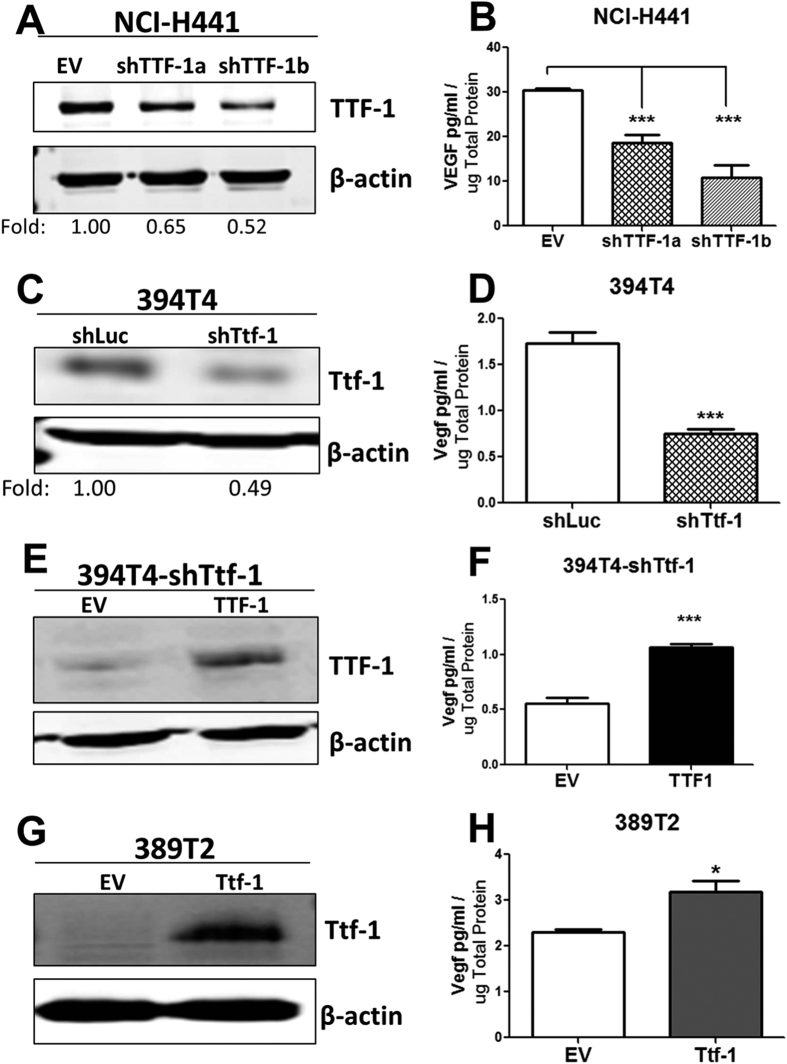
Reduction of TTF-1 expression decreases VEGF levels. Two independent shRNAs targeting *TTF-1* and the empty vector control (EV) were stably integrated into a TTF-1^+^ human lung AD cell line, NCI-H441. The reduction of endogenous TTF-1 protein was determined by the immunoblot and quantified as shown (**A**). The amount of secreted VEGF protein was quantified by an ELISA assay for the NCI-H441-based TTF-1 knockdown cells (**B**). The Ttf-1^+^ mouse lung cancer cell line 394T4 bearing a stably integrated shRNA targeting *Ttf-1* harbors a reduced expression of the endogenous Ttf-1 protein (**C**) and a decrease of Vegf protein present in the CM (**D**). To rescue Ttf-1 expression in the 394T4-shTtf-1 cells, the human wt-*TTF-1* cDNA was stably integrated into the 394T4-shTtf-1 cells. The expression of wt-TTF-1 protein was detected by western blotting (**E**) and the Vegf level in the CM increased per ELISA assays (**F**). The Ttf-1^−^ mouse lung cancer cell line 389T2 bearing a *Ttf-1* transgene expression exhibits a positive Ttf-1 protein level (**G**) and an increase of Vegf in the condition media per ELISA assays (**H**). shLuc, a control shRNA targeting the luciferase gene.

To explore how loss of TTF-1 expression would impact VEGF level, we took on two types of cell systems – (i) the human NCI-H441 cells with endogenous *TTF-1* gene amplification^3^ and (ii) the murine lung tumor cell lines (394T4 and 389T2) derived from the conditional *Kras*^*LSL-G12/+*^*/p53*^*flox/flox*^ mice^13^. Two independent small hairpin (sh) RNAs (shTTF-1a and shTTF-1b) were stably transduced into NCI-H441 cells using retroviruses to suppress endogenous TTF-1 expression (Fig. 3A). The CM was collected after a 48-hr culture in media containing 1% FBS and assessed for VEGF by ELISA. Both shRNAs conferred a significant reduction of secreted VEGF in the media (Fig. 3B). We then turned our attention to the mouse lung tumor cells isolated by Winslow *et al*.^13^. The first cell line was 394T4 which was derived from a Ttf-1^high^ primary murine lung tumor incapable of metastasizing. Upon reduction of the endogenous Ttf-1 expression in the 394T4 (Ttf-1^high^) cells using a shRNA (shTtf-1), Winslow *et al*. showed that the resultant cells (394T4-shTtf-1) gained in metastatic potential^13^. We quantified Vegf in the CM of 394T4-shTtf-1 and 394T4-shLuc (a control cell harboring a shRNA targeting luciferase). The results demonstrated a clear decrease of Vegf in the knockdown cells (Fig. 3C,D). To rescue the *Ttf-1* knockdown in the 394T4-shTtf-1 cells, we introduced the human *TTF-1* cDNA into these cells based on the fact that the shTtf-1 targeting sequence (CGCCATGTCTTGTTCTACCTT) is unique to the mouse *Ttf-1*. The expression of human TTF-1 in the 394T4-shTtf-1 cells clearly resurrected Vegf in the media by about 2-fold (Fig. 3E,F). Next, we used a second mouse lung tumor cell line (389T2) derived from a metastasizing, Ttf-1^low^ primary murine lung AD. Again, it was shown by Winslow *et al*. that the expression of a *Ttf-1* transgene in 389T2 (Ttf-1^low^) cells (Fig. 3G) suppressed cellular metastatic potential. Our ELISA measurements show that the Vegf level was higher in the CM of 389T2-Ttf-1 relative to the control 389T2-EV (Fig. 3H). Overall, the results are in line with the thesis that TTF-1/Ttf-1 positively regulates VEGF/Vegf in both human and murine backgrounds.

### VEGF Promoter Harbors Multiple TTF-1-Responsive Elements

**Figure 4:**
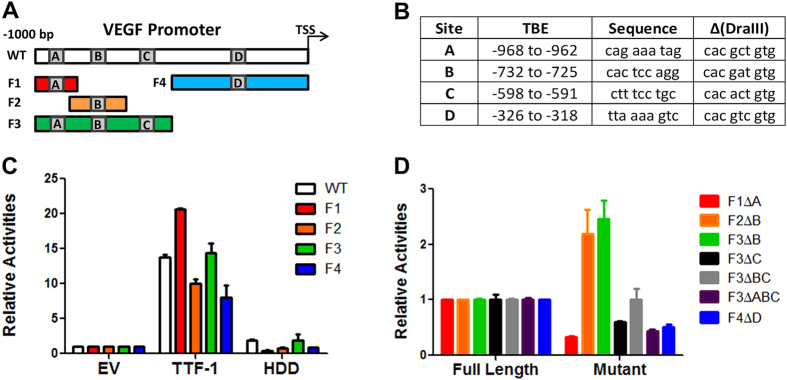
VEGF promoter contains TTF-1-responsive elements. The full-length *VEGF* promoter contains four predicted TBEs (site **A–D**). Luciferase-based reporter plasmids were constructed for the full-length *VEGF* promoter and four fragments (**A**). The locations and sequences of the four predicted TBEs are shown and each TBE was mutated to a DraIII restriction recognition sequence to test its responsiveness to TTF-1 (**B**). The relative luciferase activities were determined for the wild-type reporter constructs (**C**) and the constructs containing altered TBEs (**D**) by cotransfection of the individual reporter plasmid and a *TTF-1* expression plasmid into A549 cells.

Our observations raised the possibility that TTF-1 may directly transactivate *VEGF* transcription. We searched the *VEGF* promoter (1000 bp upstream of the TSS) for matches with chromatin immunoprecipitation/sequencing (ChIP-seq)-derived TTF-1 binding elements (TBEs)^10^,^25^. Four tentative TBEs were found (sites A–D, Fig. 4A,B). The full-length 1000-bp *VEGF* promoter was cloned into a luciferase reporter plasmid. Fragments of the full-length reporter plasmid were generated: F1 (−1000 to −800), F2 (−950 to −700), F3 (−1000 to −500), and F4 (−500 to −1) (Fig. 4A). The luciferase activity readout of each reporter plasmid was used to assess the responsiveness of each promoter to TTF-1 or the transcriptionally inactive HDD mutant of TTF-1 via transient cotransfection into A549 cells. Each of the five reporters (full-length and four fragments) scored strongly in the *TTF-1* transfected cells but not in the HDD transfected cells (Fig. 4C), implicating there may be multiple functional TBEs in the *VEGF* promoter. To test the validity of individual predicted TBEs, the TBEs within the four smaller reporter fragments were sequentially mutated to the DraIII restriction site (Fig. 4B). Promoter activity of mutant fragments was again assessed in the TTF-1 transfected A549 cells whereas both EV and HDD transfected cells demonstrated negligible promoter activity (data not shown). Reporter activity fold changes of mutated promoters were expressed as a percentage of the corresponding wild-type fragment (Fig. 4D). Alterations at the A, C, or D sites resulted in dampened signals, suggesting they are TTF-1 responsive. Surprisingly, site B appears inhibitory because the F2ΔB and F3ΔB constructs reported an increase in promoter activity. Taken together, TTF-1 appears to directly regulate VEGF expression.

### TTF-1 Also Regulates VEGFR2

**Figure 5:**
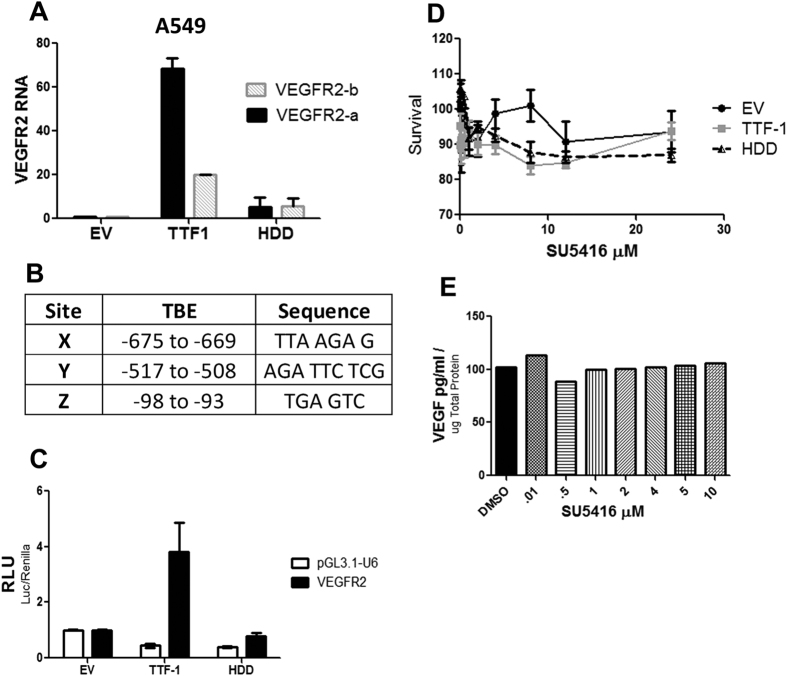
VEGFR2 is also subject to TTF-1 regulation. Transfectant A549 cells stably expressing TTF-1, HDD, or empty vector (EV) were probed by two sets of VEGFR2 primer pairs by RT-qPCR (**A**). The *VEGFR2* promoter contains three predicted TBEs (**B**). Luciferase reporter assays were conducted with a *VEGFR2* promoter containing reporter plasmid or the control reporter (pGL3.1-U6) by cotransfection with either TTF-1- or HDD-expressing plasmid into A549 cells (**C**). Three stable transfectant A549-based cells (EV, HDD, or TTF-1) were monitored for cell viability in the presence of increasing concentrations of SU5416 (**D**). Secreted VEGF was quantified in the CM of untransfected A549 cells treated with SU5416 (**E**). RLU, relative luciferase activity units.

Since a VEGF/VEGFR2 signaling autocrine loop has been found to be functionally operant in lung tumor epithelial cells^26^, we surmised that VEGFR2 may also be subject to TTF-1 regulation. To this end, we analyzed the A549 cells stably expressing *TTF-1* (or HDD) and detected elevated levels of *VEGFR2* in a TTF-1-dependnet manner (Fig. 5A). By scanning the promoter region of *VEGFR2* (−700 to +200 bp), we located three putative TBEs (Fig. 5B). A *VEGFR2* promoter reporter containing these three predicted TBEs was assayed for responses to TTF-1. The luciferase reading increased specifically in the cells transfected with TTF-1 (but not HDD) suggests that TTF-1 may also regulate VEGFR2 expression (Fig. 5C). To determine if the VEGFR2 was involved in promoting the survival of TTF-1^+^ lung cancer cells, cells were cultured in the presence of VEGFR2 inhibitors SU5416 (Fig. 5D) or SU1498 (data not shown). Cell viability (Fig. 5D) and VEGF secretion levels (Fig. 5E) were unaffected by VEGFR2 inhibitors. These data suggest that TTF-1 can modulate VEGFR2 expression and the functional significance of the autocrine signaling of VEGF/VEGFR2 in the context of TTF-1^+^ lung cancer epithelial cells is not manifested in promoting cell survival.

### Increased VEGF Secretion by TTF-1 is Partially Mediated by mTOR

VEGF production was previously demonstrated to be semi-dependent on mTOR signaling in a TSC2 dependent manner^27^. To test if the increased VEGF secretion by TTF-1 involves mTOR, A549-TTF-1 cells were treated with varying concentrations of rapamycin. VEGF secretion in the media clearly was decreased by approximately 33% at 25 nM of rapamycin (Supplemental Fig. 1A). This trend held true in the two mouse lung cancer cell systems - 389T2 transfected with a human *TTF-1* transgene (Supplemental Fig. 1B) and 394T4-shTtf-1 knockdown cells rescued with a human *TTF-1* transgene (Supplemental Fig. 1C). In all three cases, rapamycin reduced VEGF secretion by roughly 20% ~ 30%. The sustained VEGF secretion in the presence of rapamycin demonstrates an mTOR-independent activity.

### Hypoxia Doesn’t Further Increase TTF-1-induced VEGF Secretion

**Figure 6:**
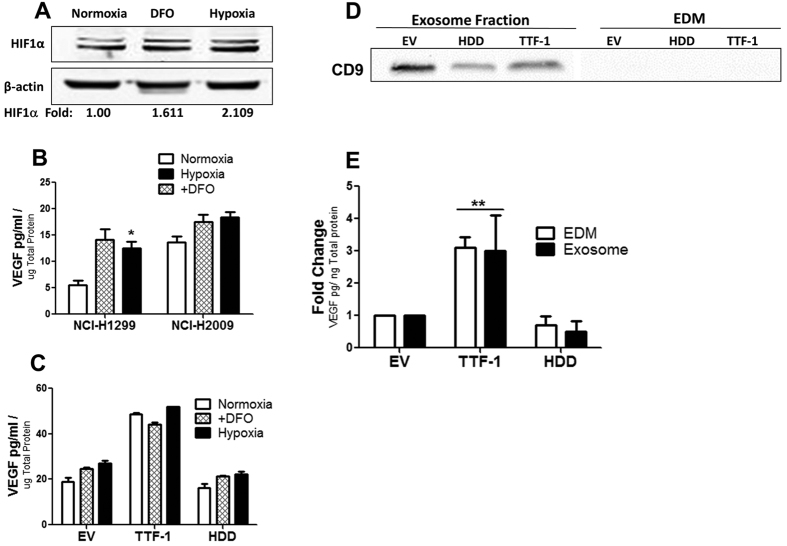
The VEGF upregulated by TTF-1 is present in exosomes in a hypoxia-independent manner. Western blots of NCI-H1299 cells subject to hypoxia or DFO show an increase of HIF1α (**A**). VEGF was quantified in the CM of NCI-H1299 (TTF-1^−^) and NCI-H2009 (TTF-1^+^) under normoxia, hypoxia, or DFO treatment (**B**). The CM of the A549 cells retrovirally transfected with *EV*, *HDD*, or *TTF-1* was analyzed by VEGF ELISA assays after three types of treatments, normoxia, hypoxia, or DFO (**C**). The exosomes and EDMs of A549-based transfectant cells were analyzed for CD9 protein expression by immunoblotting (**D**) and VEGF ELISA assays (**E**).

Hypoxia-inducible factor-1α (HIF1α) is a potent inducer of VEGF expression^28^. To determine if hypoxia would further increase TTF-1-dependent VEGF upregulation, cells were subject to hypoxia (2% O_2_) and CM was analyzed for VEGF levels. A hypoxia-mimetic, deferoxamine (DFO), was also applied under normoxic conditions as a second means to impose hypoxia-like condition^29^. To ensure hypoxic conditions were generated, the cell lysates of a TTF-1^−^ cell line (NCI-H1299) were probed for HIF1α after a 24-hr hypoxic exposure or a treatment with 200 μM of DFO. Elevated levels of HIF1α protein were detected in both treatments (Fig. 6A). Subsequently, the CM of native NCI-H1299 (TTF-1^−^) and NCI-H2009 (TTF-1^+^) cells treated with either hypoxia or DFO were assessed by ELISA. An increase in VEGF secretion was seen with both DFO and hypoxic treatment (Fig. 6B). We then treated the A549-based stable transfectant cells with either DFO or hypoxia and quantified the secreted VEGF in the media. A further increase of VEGF in the media was not observed for the cells stably overexpressing TTF-1 (Fig. 6C). Taken together, these results suggest that hypoxia doesn’t further enhance VEGF secretion of cells with TTF-1 overexpression.

### TTF-1-dependent VEGF Upregulation Occurs in Exosomes

Since exosomes have been implicated to play a role in angiogenesis^30^,^31^, we posited that the exosomes derived from the TTF-1^+^ lung cancer cells may also contain a higher level of VEGF. We used the ultracentrifugation method to isolate exosomes^32^ and the exosome-depleted fraction of the CM (i.e. EDM) from three stable transfectant cells lines, A549-EV, A549-TTF-1, and A549-HDD. By nanoparticle tracking analysis (NanoSight NS300, Malvern Instruments), the average diameter of the nanoparticles present in our exosome isolates was approximately 130 nm (data now shown), approaching the upper limit of reported size of exosomes^33^. The immunoreactivity of CD9, a canonical exosome marker^34^, was detected only in the exosome fraction and absent in the EDM (Fig. 6D). By ELISA, both fractions derived from the A549-TTF-1 cells contain a higher level of VEGF relative to the two controls (EV and HDD, Fig. 6E). These data suggest that the exosomes of TTF-1^+^ lung cancer cells also contain a higher level of VEGF.

### EDM of TTF-1^+^ Lung Cancer Cells Inhibits Angiogenesis *in vitro*

**Figure 7:**
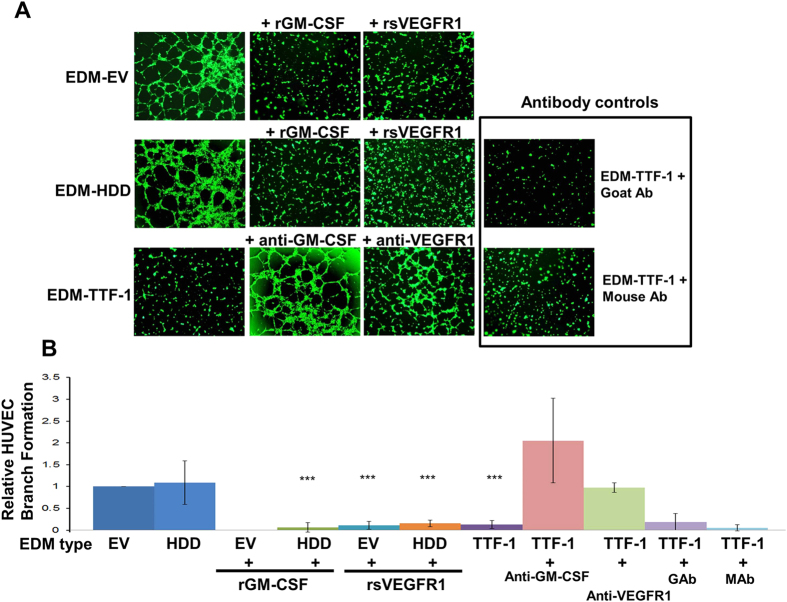
The CM of TTF-1^+^ cells confers antiangiogenic effects via the GM-CSF/sVEGFR1 axis. (**A**) Representative images of the endothelial cell tube formation assay (tube assay) assessing CMs of cells with different TTF-1 expression status plus treatments with recombinant proteins of GM-CSF/sVEGFR1 or antibodies against GM-CSF/VEGFR1. (**B**) Quantified results of branch formation of the tube assays under various conditions as in (**A**). Node formations followed the trend as observed for branch formations^a^. GAb, control goal antibody; MAb, control mouse antibody.

**Figure 8:**
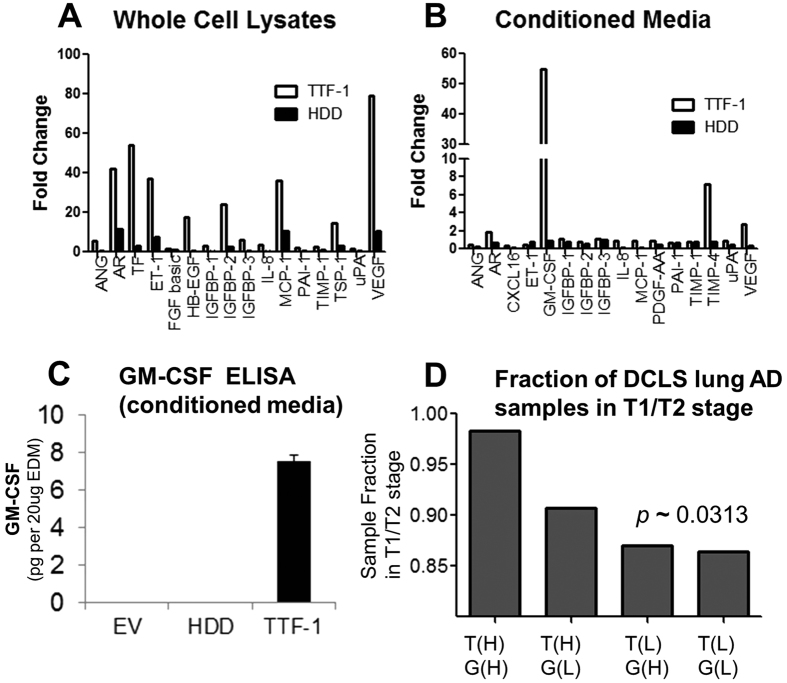
Antibody array profiling identifies GM-CSF upregulation in the CM of TTF-1^+^ cells and clinical correlations of TTF-1/GM-CSF expression. A commercial antibody array targeting 55 angiogenic proteins was used to profile cell lysates (**A**) and CM (**B**) of three types of A549 stable transfectant cells: *EV*, *HDD*, and *TTF-1*. The fold changes of protein levels were normalized to the EV control. Conditioned media derived from the A549 transfectant cells were also analyzed by a GM-CSF ELISA assay (**C**). The DCLS dataset was segregated into four groups based on the median expression values of *TTF-1* and *GM-CSF*. T(H)/G(H) denotes the subgroup with both *TTF-1* and *GM-CSF* expression values above the medians, whereas T(H)/G(L) denotes the subgroup with the *TTF-1* level above the median and the *GM-CSF* level below the median. Logistic regression for relation was used to analyze the correlation between gene expression-based sample stratification and tumor stage.

Our observation that TTF-1 increases the abundance of VEGF in the lung cancer secretome predicts that the EDM of TTF-1^+^ cells, which contains the bulk of TTF-1-induced VEGF, would likely be proangiogenic. To test this hypothesis, we isolated EDMs from three types of A549 lung AD cells stably transfected with EV, HDD, or TTF-1. The resultant EDMs are termed: EDM-EV, EDM-HDD, or EDM-TTF-1. Both EDM-EV and EDM-HDD showed positive and basal activities in the endothelial cell tube formation assay (referred to hereafter as the tube assay, Fig. 7A). Unexpectedly, EDM-TTF-1 did not score at all in the assay (Fig. 7A), suggesting the presence of angiogenesis inhibitor(s) in EDM-TTF-1. To identify such factors, we used an antibody array (Human Angiogenesis Array Kit ARY007, R&D Systems) to quantify the protein expression patterns of 55 angiogenesis-relevant factors in the three CMs and whole cell lysates. The readings of TTF-1 and HDD transfectant cells were normalized to those of the EV control cells. In the cell lysates (Fig. 8A), VEGF expression showed the highest increase at nearly 80-fold in TTF-1 cell lysates. The TTF-1 cell lysates also demonstrated an increase in Amphiregulin (AR), Coagulation Factor III (TF), Endothelin-1 (ET-1), Heparin-binding EGF-like growth factor (HB-EGF), Insulin-like growth factor binding protein 2 (IGFBP-2), Monocyte chemoattractant protein-1 (MCP-1), and Thrombospondin-1 (TSP-1). Interestingly in the CMs (Fig. 8B), only three secreted factors showed 2-fold or higher increases - GM-CSF (54-fold), Tissue inhibitor of metalloprotease 4 (TIMP-4, 7-fold), and VEGF (2.7-fold). VEGF was the only protein that was found to be increased in both cell lysates and culture media. Importantly, all the observed protein expression alterations in either cell lysates or CM of TTF-1^+^ cells were significantly diminished in the HDD transfectant cells, suggesting that the TTF-1 transcriptional activity was responsible for the observed expression alterations. The strong upregulation of GM-CSF in CM was validated by a GM-CSF specific ELISA assay (Fig. 8C). GM-CSF is known to induce soluble VEGFR1^35^,^36^ which confers antiangiogenic activities by sequestering VEGF from interacting with signaling VEGFRs on the cell surface^37^. To test the candidacy of GM-CSF as a factor mediating the antiangiogenic activity of EDM-TTF-1, we used the tube assay to assess the angiogenic potential of the three EDMs with various treatments. The results indicate that recombinant proteins of GM-CSF (rGM-CSF) or sVEGFR1 (rsVEGFR1) robustly suppressed the endothelial tube formation capacity of EDM-EV and EDM-HDD (Fig. 7A and B), whereas the treatment of anti-GM-CSF or anti-VEGFR1 antibodies restored the tube formation activity of EDM-TTF-1 (Fig. 7A,B). By qRT-PCR, *GM-CSF* RNA was increased quantitatively in the *TTF-1*-transfected cells (data not shown). These data are consistent with the notion that GM-CSF is subject to TTF-1 regulation and that TTF-1 putatively reprograms the CM into an antiangiogenic state through the GM-CSF/sVEGFR1 axis.

### RNA Expression Correlations of TTF-1, VEGFA, VEGFR1, VEGFR2, and GM-CSF in Human Lung ADs

To explore the expression relationship between *TTF-1*, *VEGFA, VEGFR1, VEGFR2, and GM-CSF* in human lung ADs and to avoid treatment-induced gene expression perturbation, we first analyzed the RNA expression data of the Director’s Challenge Lung Study (DCLS)^38^ which comprises gene expression profiles of over 400 early-stage lung ADs without preoperative chemotherapy or radiation. As shown in Supplemental Table 1, positive and statistically significant correlations were found for these gene pairs: *TTF-1/VEGFR2*, *VEGFA*/*VEGFR2*, *TTF-1*/*GM-CSF*, *TTF-1*/*VEGFR1*, and *GM-CSF*/*VEGFR1*. Intriguingly, the *GM-CSF/VEGFR1* correlation was the most positive (r = 0.4101, *p* < 0.0001). The correlation between *TTF-1* and *VEGFA* was weakly positive (r = 0.08722) and less statistically significant (*p* = 0.0785), perhaps a reflection of the complexity of *VEGF* regulation *in vivo*. The expression between *VEGFR1* and *VEGFR2* was the only statistically significant relationship in the negative direction (r = −0.17353, *p* = 0.0004). We then moved to a smaller gene expression profiling study of human lung ADs (GSE42127)^39^ and only analyzed the data derived from those samples without adjuvant chemotherapy. In this smaller dataset (Supplemental Table 2), a positive correlation between *TTF-1* and *VEGFR2* was observed (r = 0.28723, *p* = 0.005), and the correlation of *GM-CSF*/*VEGFR1* remains positive (r = 0.2317, *p* = 0.0214). Finally, by segregating the DCLS data into four groups based on the median expression values of *TTF-1* and *GM-CSF*, we noticed that the (*TTF-1*(high)/*GM-CSF*(high), T(H)/G(H)) group was most enriched in earl-stage lung ADs (98.2% in T1 + T2, Fig. 8D). These results of expression correlations derived from human lung ADs are largely consistent with our cell-based observations in this study.

## Discussion

Our interest in exploring how TTF-1 modulates the proteinaceous secretome of lung cancer cells motivated us to initiate this study by first conducting a focused qPCR profiling of cytokine expression between TTF-1-on/off states. Among the significantly upregulated cytokines in the TTF-1-on state, *BMP4* was already a known transcriptional target of TTF-1^40^. Subsequently, we chose to further characterize the regulatory connection between *TTF-1* and *VEGF* for these reasons: (i) *Vegf* expression perturbation occurs in *Ttf-1* mutant mice^22^,^23^, (ii) *VEGF* was not established as a direct transcriptional target of *TTF-1*, (iii) VEGF is a master regulator of angiogenesis^24^, and (iv) Our focused angiogenesis factor expression profiling detected an overall trend of angiogenic factor upregulation by TTF-1 (Fig. 1B), suggesting a proangiogenic phenotype associated with the TTF-1-on state. In both gain- and loss-of-TTF-1 expression studies, we establish that VEGF/Vegf levels in the media fluctuate with TTF-1/Ttf-1 expression changes, supporting a positive regulation of VEGF by TTF-1. Indeed, four TBEs were identified in the *VEGFA* promoter. Based on the luciferase reporter assays, three of the four TBEs appear to be responsive to TTF-1 and mediate the positive regulation of *VEGF* transcription by TTF-1. However, surprisingly site B in the *VEGF* promoter conferred an increased luciferase reporter activity when deleted, suggesting that site B mediates a repressive regulation of the *VEGF* promoter. Our data do not rule out the existence of additional cryptic TBEs in the *VEGF* promoter. Nevertheless, our findings of functional TBEs present in the *VEGF* promoter are in line with the notion that *VEGF* is a direct transcriptional target of TTF-1. We note that in ChIP-seq studies conducted in the lung cancer cell line A549^10^ and NCI-H661^41^, genomic occupancies of TTF-1 were found to be near *VEGFA*. This further corroborates our data reported herein.

We next turned our attention to a major signaling receptor of VEGF, i.e. VEGFR2, in our lung cancer epithelial cell systems. This move was inspired by the accumulating evidence that the autocrine VEGF:VEGFR signaling of tumor epithelial cells functionally contributes to cancer initiation and maintenance^26^,^42^,^43^,^44^. Indeed, we observed that TTF-1 appears to increase the transcription of *VEGFR2* and the *VEGFR2* promoter reporter was responsive to wt-TTF-1 but not to the HDD mutant of TTF-1. However, the VEGFR2 inhibitor SU5416 did not suppress the proliferation of the A549-TTF-1, nor did SU5416 inhibit the VEGF secretion in the A549-TTF-1 cells. These data imply that the functional consequences of the VEGF/VEGFR2 autocrine signaling lie beyond proliferation for the A549-TTF-1 cells and that VEGFR2 is not involved in the increased VEGF secretion in our cell systems. This is in contrast with the observation of Chatterjee *et al*. that VEGFR2 is involved in upregulating VEGF secretion in lung cancer cells^26^.

A previous study identified a Ttf-1-related protein expression signature in the plasma of mice with lung ADs^45^. However, how TTF-1 modulates exosomal protein cargoes was completely unknown. We thus isolated exosomes from the CMs of A549-EV, A549-HDD, and A549-TTF-1 cells using multiple rounds of ultracentrifugation. We knew that the bulk of the TTF-1-induced increase of secreted VEGF must be present in the EDM because exosomes only represent a tiny portion of the whole condition media. However, we did not know whether the exosomes of A549-TTF-1 cells would also contain a higher level of VEGF. By ELISA, it was clear that exosomal VEGF concentration was proportionally increased as in EDM, thus establishing the first example of TTF-1 modulating an exosomal cargo protein. At this juncture, we are profiling the exosomal proteome of A549-TTF-1 and control cells to quantify the extent of the influence of TTF-1 on exosomal proteome. We consider the identification of exosomal VEGF level subject to TTF-1 control a piece of evidence that there are other exosomal protein cargoes also directly or indirectly regulated by TTF-1. Exosomal cargoes include nucleic acids such as microRNAs (miRNAs)^46^,^47^. In light of our discoveries of miRNAs interacting with *TTF-1*^14^,^15^, it will be interesting to determine whether exosomal miRNA profiles are also under TTF-1 regulation.

One of the most surprising findings in our study is the apparent inhibitory activity of EDM-TTF-1 in the tube assay. To resolve this puzzle, we profiled both cell lysates and CMs of EV, HDD, and TTF-1 transfectant cells using an antibody array targeting 55 angiogenic factors. The strong, TTF-1-dependent upregulation (>50X) of secreted GM-CSF prompted us to further investigate the contribution of the GM-CSF/sVEGFR1 to the tube assay inhibitory phenotype of EDM-TTF-1 because GM-CSF has been found antiangiogenic by way of sVEGFR1 upregulation. GM-CSF is known to induce sVEGFR1^35^,^36^ which confers antiangiogenic activities by sequestering VEGF from interacting with canonical signaling VEGFRs on the endothelial cell surface^37^. Using both gain- and loss-of-function types of tools – antibodies against GM-CSF or VEGFR1 and recombinant proteins of GM-CSF and sVEGFR1, we generated data consistent with the notion that the GM-CSF/sVEGFR1 axis mediates the antiangiogenic activities of EDM-TTF-1. The *GM-CSF* (or known as *CSF2*) RNA upregulation by TTF-1 was actually detected in our original qPCR-based cytokine profiling at approximately 2-fold (Fig. 1A), consistent with the idea that *GM-CSF* is a direct transcription target of TTF-1. Indeed, analysis of *GM-CSF* promoter identified minimally two putative TBEs and the *GM-CSF* RNA was elevated in the *TTF-1*-overexpressing cells by qRT-PCR (data not shown). We note that the anti-VEGFR1 antibodies did not completely restore the tube assay positivity of EDM-TTF-1 to the level of the experiment of EDM-TTF-1 plus anti-GM-CSF antibodies (Figs. 7A and 7B). These data suggest that sVEGFR1 may not be the sole mediator of GM-CSF-dependent antiangiogenic activities in our systems. Our current thesis regarding the source of sVEGFR1 in our experimental tube assay system is that it originates, in a paracrine fashion, from the HUVECs which secrete sVEGFR1 upon stimulation by the TTF-1^+^ tumor cell-derived GM-CSF. Future work will shed light on this issue. The EDM-induced phenotypes in the tube assay occurred over a time period of 2 ~ 3 hr. Considering that the doubling time of HUVECs being 48 hr^48^, we did not detect an alteration in the absolute HUVEC number before and after the tube assay (data not shown). Therefore, the EDM-prompted effects on HUVECs appear to be specific to branching/tube formation.

By examining the DCLS lung AD gene expression profiling data consisting of 408 lung ADs without preoperative treatment, we detected multiple statistically significant and positive correlations of these gene pairs: *TTF-1*/*VEGFR2*, *VEGFA*/*VEGFR2*, *TTF-1*/*GM-CSF*, and *GM-CSF*/*VEGFR1*. These observations largely reinforce our cell-based observations. In particular, the positive correlation of *GM-CSF*/*VEGFR1* persisted statistically in another smaller lung AD gene expression dataset (GSE42127). Moreover, our observations documented herein, including that *TTF-1*(high)/*GM-CSF*(high) subgroup of the DCLS dataset appears enriched in early T1/T2 stage of lung ADs, are seemingly in line with many studies reporting that lung ADs with TTF-1 immunopositivity have better clinical outcome^49^,^50^,^51^,^52^,^53^. However, at this juncture we do not know if the enrichment of TTF-1^+^ lung ADs in T1/T2 stages is sufficient to account for the better clinical outcome of TTF-1^+^ lung ADs. Future studies to contrast the prognosis of the *TTF-1*(high)/*GM-CSF*(high) vs *TTF-1*(low)/*GM-CSF*(low) subgroups within individual stages of human lung ADs would provide a deeper understanding regarding the clinical correlation of TTF-1 and GM-CSF expression status.

In light of our findings, it is tempting to suggest that the antiangiogenic secretome of TTF-1^+^ lung cancer cells may contribute to the well-documented, better clinical outcome of TTF-1^+^ lung ADs. However, there is ample evidence implicating that primary lung ADs tumors could progress without angiogenesis^54^. Such a “vascular co-option” mechanism would allow tumors to obtain a blood supply by hijacking the existing vasculature^55^. Perhaps, certain TTF-1^+^ lung ADs may be primed to undergo vascular co-option for growth and maintenance in a manner independent of angiogenesis. In summary, we take the data reported herein as the evidence that TTF-1 may reprogram the function of lung cancer secretome. In view of the known roles of GM-CSF in modulating immune cells^56^, it is expected that future work will also be directed at understanding how TTF-1 reprograms the immunomicroenvironment of lung ADs.

## Methods

### Cell Culture and Expression Vectors

The human lung cancer cell lines A549, BEAS-2B, NCI-H441 (H441), NCI-H1299 (H1299), NCI-H1792 (H1792), and NCI-H2009 (H2009) cells were acquired from the American Type Culture Collection (ATCC), and maintained per the recommendation of ATCC. Human lung cancer cell line HCC44 was obtained from the Leibniz Institute (DSMZ) and maintained in RPMI-1640 supplemented with 10% fetal bovine serum (FBS), penicillin and streptomycin. Human umbilical vein endothelial cells (HUVEC) were obtained from either Fisher Scientific (#8774388) or Life Technologies (#C-003-5C) and grown in Media 200 supplemented with low serum growth supplements (Life technologies, #S-003-10). Mouse 394T4-bc37 (shLuc), 394T4-E1 (shTtf-1), 389T2-EV, and 389T2-Ttf-1 cells were provided by Dr. Monte Winslow and maintained in DMEM supplemented with 10% FBS, penicillin and streptomycin. Two shRNAs targeting *TTF-1*, based on Weir *et al*.^6^, were inserted into the MSCV/LTRmiR30-PIGΔARI (EV) retrovirus vector. The pcDNA3.1-based *TTF-1* and *TTF-1* homeodomain deletion mutant (HDD) expression vectors were previously constructed^11^,^15^. The *VEGF* (−1000 to 0, relative to Transcription start site (TSS)) promoters were PCR-amplified from human genomic DNA using primers listed in Supplemental Table 3 and cloned into the luciferase vector pGL4.10 (Promega, #E6651). The generated plasmid was used as a template to create smaller reporter fragments with primers listed in Supplemental Table 3. Mutant *VEGF* promoter reporters were generated using the Quick Change II XL Site-Directed Mutagenesis kit (Agilent, #200521) according to manufacturer’s protocol; the primers are included in Supplemental Table 3. The F1ΔA and FΔABC vectors were created by PCR-amplification of g-Blocks (Integrated DNA Technologies) for the specific *VEGF* promoter sequence containing DraIII substitution at the listed sites. The pGL3-mVEGFR2-780 (−780 to +268, relative to TSS, #51133) and pGL3-U6-sgRNA-PGK-puro (Control, #21307) were acquired from Addgene.

### Transfections

Stable transfectant cell lines were generated using the Lipofectamine 2000 (Life Technologies #11668019) according to manufacturer’s protocol. Doxycycline (Dox)-inducible systems (BEAS-2B, HCC44, and NCI-H1792) were created by transfecting cells followed by G418 (VWR International #45000-630) selection. Stable cell lines were created by transfecting cells and selecting with puromycin (A549) or hygromycin (394T4-TTF-1) (VWR International #45000-806). Luciferase promoter transfections were conducted using the K2 Transfection System (Biontex #T060-8.0) or Lipofectamine 2000 (*VEGFR2*).

### RNA Isolation and Reverse-Transcription (RT)-Quantitative PCR (QPCR)

RNA was isolated from cells using TRIzol (Life Technologies #15596-018) and Direct-zol RNA MiniPrep kit (Zymo Research #R2052). For mRNA quantification, RNA was reverse transcribed using the EasyScript cDNA Synthesis Kit (Applied Biological Materials #G234). The generated cDNA was evaluated by Real-time PCR using Eva Green 2X PCR master mix-ROX (Applied Biological Materials #3538) with a StepOne Plus Real-Time PCR System (Applied Biosystems).

### PCR Arrays

Qiagen arrays were used to examine 84 genes related to cytokines (PAHS-021Z) or angiogenesis (PAHS-024Z) in the BEAS-2B-based dox-inducible system according to manufacturer’s instruction. RNA was isolated and cDNA was generated using the RT^2^ First stand kit (Qiagen #330401). Expression levels were measured using RT^2^ SYBR Green qPCR Master Mix (Qiagen #330520) with a StepOne Plus Real-Time PCR System (Applied Biosystems).

### Antibody Array

Fifty-five angiogenesis-related proteins were profiled in the cell lysates and CM of the A549 transfectant cells carrying an stable integration of the *TTF-1* transgene using an antibody array following manufacturer’s suggestion (R&D Systems #ARY007). Briefly, following that cells were grown in RPMI-1640/1% FBS for 48 hours, conditioned media was collected and cells were lysed with lysis buffer (1% NP-40, 20 mM Tris-HCl pH 8, 137 mM NaCl, 10% glycerol, 2mM EDTA, and 1X Halt protease inhibitors (Fisher Scientific #PI87785)). Concentrations of protein samples were determined by Bicinchoninic Acid assay (BCA) (Thermo Scientific #23225). Array membranes were incubated with either 200 μg of cell lysate or 500 μg of conditioned media. Membranes were developed on X-ray film (GE Healthcare #95017-659); densities of each spot was determined using UN-SCAN-IT (Silk Scientific).

### Western Blotting

Total cell lysates were harvested in RIPA buffer with Halt protease inhibitor cocktail. Concentration was measured by BCA. Cell lysates (15–30 μg) were fractionated by SDS-PAGE (BioRad System) and electrophoretically transferred to nitrocellulose membranes (VWR International #74330-014). Membranes were blocked in 5% bovine serum albumin (Cell Signaling, #9998S) Tris-buffered saline and incubated overnight with primary antibodies: TTF-1 1:500 (sc-H190, Santa Cruz Biotechnologies #SC-13040), HIF1α 1:500 (clone 16H4L13, Fisher Scientific #70050), VEGF 1:500 (sc-152, Santa Cruz Biotechnology), CD9 1:1000 (D801A, Cell Signaling Technologies, #13174) or β-actin 1:20,000 (Cell Signaling, mouse #3700P, rabbit #4970P). Proteins were detected with fluorescent secondary antibodies 1:20,000 (Licor, IRDye 700/800, anti-mouse #926-68070, anti-rabbit #926-32211) on an Odyssey Infrared Imager (Licor).

### ELISA

Concentrations of protein samples were determined by BCA. Conditioned media collected from human cells was concentrated with centrifugal 10-kDa filters of various sizes (Fisher Scientific #UFC201024, UCF801008, UCF201024) to enhance detection. VEGF ELISA coated wells were loaded with identical amounts of total proteins, and assay was conducted according to manufacturer’s instructions (RayBiotech #ELH-VEGF-001). Absorbance was measured on a H2 Synergy (BioTek) at 450 nm. Cells were grown in the presence of rapamycin (LC Laboratories, #R-5000) for 24 hours before conditioned media was collected. The GM-CSF ELISA assay was conducted in a similar fashion per manufacturer’s protocols (RayBiotech #ELH-GMCSF).

### Luciferase Reporter Assay

Promoter reporter assays were carried out in 24-well plates using A549 parental cells as previously described^11^,^15^. Briefly, cells were co-transfected with Firefly luciferase reporter construct and *Renilla* luciferase control vector pGL4.10 (Promega). Firefly and *Renilla* luciferase values were measured 48 hours after transfection using Firefly and Renilla Dual Luciferase Assay Kit (Biotium #30005-1) or Dual-Glo luciferase assay (Promega #PAE2920) on a GloMax 96 plate reader (Promega). *Renilla* luciferase signals were normalized to Firefly luciferase signals.

### Cell Viability

The effect of VEGFR2 inhibitor SU5416 (Sigma #S8442-5MG) on cell viability was tested in A549 cells. Cell survival was assessed 72 hours after treatment using Cell Titer Blue (Promega, #PAG8080) according to manufacturer’s instruction. Absorbance was read 2 hours after addition on a BioTek H2 Synergy spectrophotometer at 560/590 nm.

### Hypoxia Treatment

Cells were grown in 60-mm dishes and placed into Nalgene containers (VWR International, #16129-414) which had been modified with holes as described previously^57^. Briefly, dishes were placed within chamber and flushed with low oxygen gas (2% O_2_, 7% CO_2_, 91% N_2_, Airgas National Welders) for 3 min, and flushed again after an initial 45 min incubation. Vacuum grease (Thomas Scientific, #8690B20) was used to seal the top and rubber stoppers were used to plug the holes of the Nalgene containers.

### Exosome Collection

Exosomes were harvested using an ultracentrifugation technique^32^. Briefly, cells were plated onto 150-mm dishes and allowed to reach approximately 90% confluency. Cells were washed 3× with PBS and afterwards exosome depleted media at 1% FBS in RPMI 1640 was added to the cells, allowing to incubate for 48 hours. The media was collected and centrifuged for 10 min at 500 g and then 20 min at 2,000 g to remove cells and debris and followed up with 20,000 g for 30 min to remove larger vesicles. The supernatant was then spun at 100,000 g for 1.5 hours to pellet the exosomes. The pellet was then washed with PBS and respun at 100,000 g for 1.5 hours again. The pellet was then resuspended in RIPA lysis buffer with Halt protease inhibitors.

### Endothelial Cell Tube Formation Assay (tube assay)

Basement membrane (Fisher Scientific, CB40234A) was plated in μ-slides (ibidi, 81506) at a concentration of 5 mg/mL and allowed to solidify for at least 30 min at 37 °C. Following a 45-min staining with 5 μg of Calcein-AM (Fisher Scientific, C3100MP) in 60-mm dishes, HUVECs were collected by trypsinization, resuspended in RPMI +10% FBS, and seeded into the Angiogenesis μ-slide at a density of 10,000 cells/50 μL of solution. A549 conditioned media was collected and removed of exosomes by centrifugation, as described above. Conditioned media, frozen at −80 °C, was thawed and concentrated using the Amicon 3K centrifugal filters (EMD Millipore). Concentrated conditioned media was analyzed for protein concentration by BCA assay. Protein concentrations between 1.5 mg/mL and 3 mg/mL were used in the tube assay. Protein concentrations were equal across treatment groups intra-experimentally. Recombinant human GM-CSF protein (250 ng/mL, Peprotech, 300-03) or sVEGFR1 protein (20 ng/mL, RayBiotech, ELH-VEGFR1) was added to the tube assay experiments to inhibit the angiogenicity of EDM-EV or EDM-TTF-1 (EDM, exosome-depleted media). Antibody concentrations of 2.0 μg/mL were used for all antibody treatments in the tube assay. Anti-GM-CSF antibody: monoclonal mouse IgG1 clone 3209 (R&D Systems, MAB215). Anti-VEGFR1 antibody: polyclonal goat IgG (R&D Systems, AF321). Mouse Ab: monoclonal mouse IgG1 clone 11711 (R&D Systems, MAB002). Goat Ab: polyclonal goat IgG (R&D Systems, AB-108-C). HUVECs were incubated for 2–3 hours at 37 °C. Images were taken on a Nikon Eclipse TS100 microscope at 40X. Images were analyzed for branch and node formation using ImageJ software^58^. Branch and node formations were normalized to controls.

### Statistics and Analysis of Gene Expression Datasets

T-test was used to compare two groups and ANOVA was used to compare multiple groups of data. Data were considered statistically significant when *p* < 0.05 (*, *p* < 0.05; **, *p* < 0.01; ***p < 0.001). Raw Affymetrix expression data of the Director’s Challenge Lung Study (DCLS) were downloaded from NCI and the CEL files imported into the Partek GS 6.6 platform (Partek Inc). RMA-normalized log signal values were compared in histograms and boxplots to discover and exclude possible technical outliers, whose signal profiles differed radically from the rest and would contribute noise to the RMA quantile normalization process. Those samples that demonstrated consistent profiles were then re-imported and normalized together to minimize batch differences among the multiple experimental datasets. These log_2_ normalized probeset signal values were used for subsequent expression analyses. The GSE42127 dataset was obtained from the GEO database and processed in a similar fashion. The error bars presented in the figures are standard errors.

## Additional Information

**How to cite this article**: Wood, L. W. *et al*. Thyroid Transcription Factor 1 Reprograms Angiogenic Activities of Secretome. *Sci. Rep*. **6**, 19857; doi: 10.1038/srep19857 (2016).

## Supplementary Material

Supplementary Information
