# Supplementary material for: Thyroid Transcription Factor 1 Reprograms Angiogenic Activities of Secretome
Source: Sci Rep. 2016 Feb 25;6:19857. doi: 10.1038/srep19857 (PMC4766481; doi:10.1038/srep19857)
Supplement: Supplementary Information [file srep19857-s1.pdf]

Supplemental Material

Thyroid Transcription Factor 1 Reprograms Angiogenic Activities of Secretome

Lauren W. Wood[1,2], Nicole I. Cox[1,2], Cody A. Phelps[1,2], Shao-Chiang Lai[1,2], Arjun Poddar[1], Conover Talbot Jr.[3], and David Mu[1,2]

[1]Leroy T. Canoles Jr. Cancer Research Center, Eastern Virginia Medical School, Norfolk, VA 23501
[2]Department of Microbiology and Molecular Cell Biology, Eastern Virginia Medical School, Norfolk, VA 23501
[3]Institute for Basic Biomedical Sciences, The Johns Hopkins School of Medicine, Baltimore, MD 21205

To whom correspondence should be addressed: David Mu, Leroy T. Canoles Jr. Cancer Research Center, Department of Microbiology and Molecular Cell Biology, Eastern Virginia Medical School, Norfolk, VA 23501 USA. E-mail: mud@evms.edu

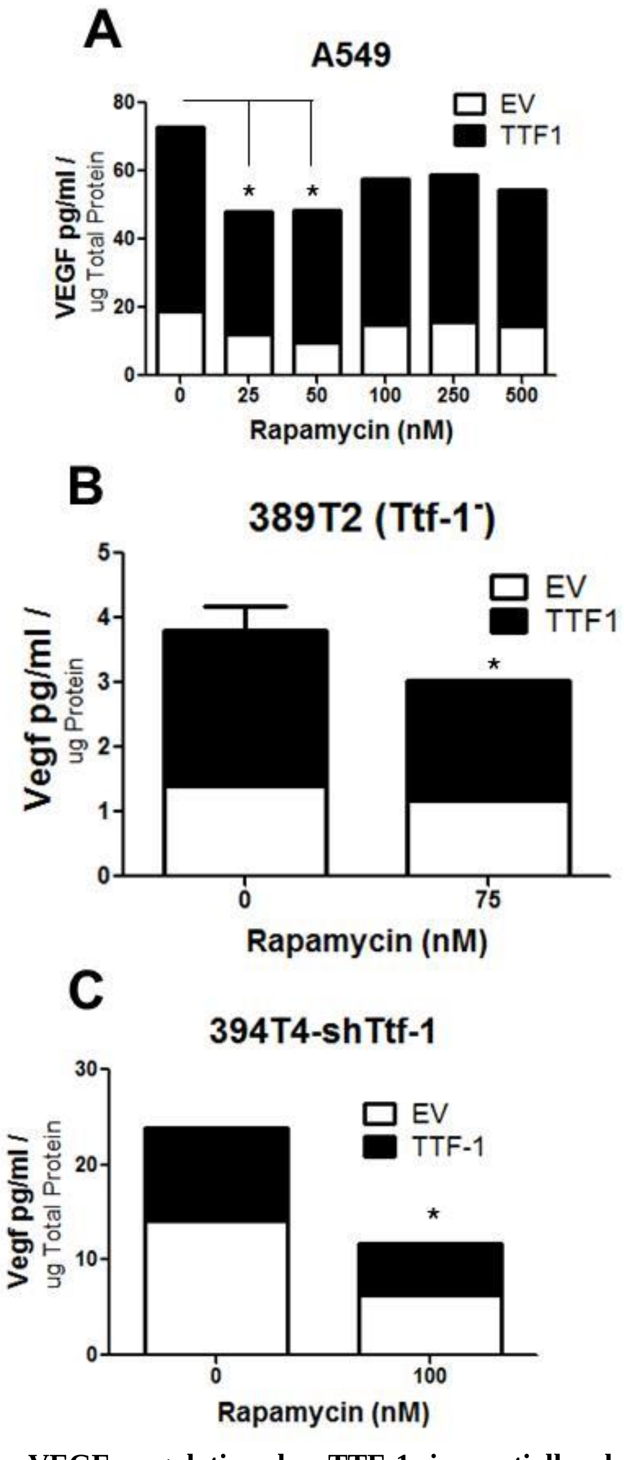

Supplemental Figure 1. **VEGF regulation by TTF-1 is partially dependent on mTOR**. The concentrations of VEGF/Vegf were quantified by ELISA assays in the CM of three types of retroviral transfectant cells with rapamycin treatments: (**A**) A549, (**B**) 389T2 without endogenous *Ttf-1* expression, and (**C**) 394T4-shTtf-1. EV, empty vector.

Supplemental Table 1: Expression correlations in the DCLS dataset (408 lung ADs).

TTF-1	TTF-1	VEGFA	VEGFR1	VEGFR2	GM-CSF
TTF-1	1	0.08722 (0.0785)	0.10007 (**0.0434**)	**0.21358 (<0.0001)**	**0.18541 (0.0002)**
VEGFA		1	-0.05244 (0.2906)	**0.30174 (<0.0001)**	0.07341 (0.1388)
VEGFR1			1	**-0.17353 (0.0004)**	**0.4101 (<0.0001)**
VEGFR2				1	-0.07806 (0.1154)
GM-CSF					1

Pearson correlation coefficients shown with the *p* value in parenthesis.

Supplemental Table 2: Expression correlations in a subset of the GSE42127 dataset (94 lung ADs without ACT).

TTF-1	TTF-1	VEGFA	VEGFR1	VEGFR2	GM-CSF
TTF-1	1	-0.01341 (0.898)	0.0974 (0.3503)	**0.28723 (0.005)**	-0.14906 (0.1516)
VEGFA		1	-0.09041 (0.3861)	0.20186 (0.0511)	0.13686 (0.1884)
VEGFR1			1	0.02461 (0.8138)	**0.2371 (0.0214)**
VEGFR2				1	-0.12854 (0.217)
GM-CSF					1

Pearson correlation coefficients shown with the *p* value in parenthesis.
ACT, adjuvant chemotherapy

Supplemental Table 3: DNA oligonucleotides used for this study

Use	Name	For/Rev	Sequence 5' → 3'
qRT- PCR			
	GAPDH	F	GGAGTCAACGGATTTGGTCGTA
		R	GGCAACAATATCCACTTTACCAGAGT
	mGapdh	F	GCTTGTCATCAACGGGAAGC
		R	TTTGATGTTAGTGGGGTCTCGC
	β-actin	F	GTTGTCGACGACGAGCG
		R	GCACAGAGCCTCGCCTT
	mβ-actin	F	ATGGAGGGGAATACAGCCC
		R	TTCTTTGCAGCTCCTTCGTT
	TTF-1 set 1	F	GCTCATGTTCATGCCGCT
		R	ACCAGGACACCATGAGGAAC
	TTF-1 set 2	F	CTCATGTTCATGCCGCTC
		R	GACACCATGAGGAACAGCG
	TTF-1 set 3	F	CCATGTTCTTGCTCACGTCC
		R	AGGAACAGCGCCTCTGG
	mTtf-1 set 1	F	GTGCTTTGGACTCATCGACA
		R	GTCCTCGGAAAGACAGCATC
	mTtf-1 set 2	F	AAAACTGCGGGGATCTGAG
		R	TGCTTTGGACTCATCGACAT
	VEGF set 1	F	CGACGGCTTGGGGAGATTGC
		R	GGGCGGTGTCTGTCTGTCTG
	VEGF set 2	F	TGGATCCATGAACTTTCTGCTGTC
		R	TCACCGCCTTGGCTTGTCACAT
	VEGF set 3	F	AGCTGCGCTGATAGACATCC
		R	CTACCTCCACCATGCCAAGT
	VEGF set 4	F	CACACAGGATGGCTTGAAGA
		R	AGGGCAGAATCATCACGAAG
	mVegf set A&B	F	AATGCTTTCTCCGCTCTGAA
		R1	GCTTCCTACAGCACAGCAGA
		R2	CTCACCAAAGCCAGCACATA
	VEGFR1 set 1	F	TCCCTTCCTTCAGTCATGTGT
		R	AAGAAGGAAACAGAATCTGCAA
	VEGFR1 set 2	F	GAGCTCCCTTCCTTCAGTCA
		R	GAAGAAGGAAACAGAATCTGCAA
	VEGFR2 Set 1&2	F	CGGCTCTTTCGCTTACTGTT
		R1	CCTGTATGGAGGAGGAGGAA
		R2	TCTCTCTGCCTACCTCACCTG
Cloning			
	hVEGFprom	F	TTAAGGTACCCCAGTCCCAAATATGTAGCTGTT
		R	TTAAAAGCTTCCACAGTGATTTGGGGAAGTA
